# Role of Hippocampal Glutamatergic Synaptic Alterations in Sevoflurane‐Induced Cognitive Dysfunction in Aged Mice

**DOI:** 10.1111/cns.70093

**Published:** 2024-10-28

**Authors:** Yixuan Niu, Guoying Liao, Zhengjie Miao, Jinnan Xu, Yanyong Cheng, Fan Wang, Chuanyu Qi, Tiannan Chen, Yi Gao, Lei Zhang, Hong Jiang, Jia Yan

**Affiliations:** ^1^ Department of Anesthesiology, Shanghai Ninth People's Hospital Shanghai Jiao Tong University School of Medicine Shanghai China; ^2^ Shanghai Jiao Tong University School of Medicine Shanghai China; ^3^ Department of Anesthesiology The Affiliated Hospital of Qingdao University Qingdao China

**Keywords:** aging, anesthesia, excitatory neuron, glutamatergic synapse, PND, sevoflurane

## Abstract

**Aims:**

Perioperative neurocognitive disorders (PND), including postoperative delirium (POD) and postoperative cognitive dysfunction (POCD), are common following anesthesia and surgery in older patients and significantly increase morbidity and mortality. However, the underlying mechanism of PND is unclear. Our study aims to analyze the differentially expressed genes (DEGs) in excitatory neurons and investigate the role of hippocampal glutamatergic synaptic alterations in sevoflurane‐induced cognitive dysfunction in aged mice.

**Methods:**

We performed single‐nucleus RNA sequencing (snRNA‐seq) technology to examine the alterations of excitatory neurons in hippocampus induced by sevoflurane in aged mice. Gene Ontology (GO) and Kyoto Encyclopedia of Genes and Genomes (KEGG) analysis of DEGs were performed in excitatory neurons. At last, immunofluorescence staining was used to validate sevoflurane‐induced alternation of glutamatergic synapses in the hippocampus of aged mice.

**Results:**

This study demonstrates that DEGs in excitatory neurons are associated with reduction of glutamatergic synapses and cognitive dysfunction. After immunofluorescence staining validation, we also confirmed that sevoflurane anesthesia decreased the density of glutamatergic synapses in the hippocampus of aged mice.

**Conclusions:**

Our findings demonstrated a key role of hippocampal glutamatergic synaptic alterations in sevoflurane‐induced cognitive dysfunction in aged mice.

## Graphical abstract

Our study in aged mice unveils sevoflurane‐induced changes in gene expression within excitatory neurons, which is associated with synaptic function and cognitive impairment. Immunofluorescence staining confirms reduced hippocampal glutamatergic synapses, emphasizing their central role in age‐related cognitive dysfunction.

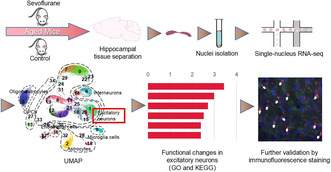

## 1 Introduction

PND, including POD and POCD, are common following anesthesia and surgery in older patients and significantly increase morbidity and mortality [1, 2, 3]. Elderly patients are particularly susceptible to experiencing prolonged memory deterioration and impaired information processing, contributing to a substantial economic burden attributed to restricted daily activities and an increased likelihood of dementia [4]. However, the underlying mechanism of PND is unclear.

Excitatory neurons, as a major neuron type in the brain and one of the most important integral components of the central nervous system, play a key role in regulating neural circuitry and cognitive functions [5]. Characterized by their ability to transmit excitatory signals through the release of neurotransmitters, particularly glutamate, excitatory neurons contribute significantly to synaptic communication within neural networks [6]. Evidence suggests that anesthesia‐induced alterations in the function and integrity of excitatory neurons may be implicated in various neurodegenerative conditions [7]. However, the mechanisms by which dysfunctions in excitatory neurons lead to cognitive dysfunction remain to be investigated.

The principal synaptic type of excitatory neurons comprises glutamatergic synapses [6]. Glutamatergic synapse serves as fundamental information processing units within the brain, with their precise numbers and specific connections being indispensable for the normal functioning of the central nervous system [6]. Specifically, glutamatergic synapses play a crucial role in processes such as memory, learning, and emotional regulation [8]. A previous study has shown that decreased hippocampal glutamatergic synapse density is associated with cognitive dysfunction [8]. Neurodegenerative disorders are primarily characterized by significant structural and functional alterations in excitatory glutamatergic synapses in the brain, leading to numerous synaptic deficits and abnormal synapse loss [7, 9]. Chen et al. [6] demonstrated that promoting glutamatergic synaptogenesis in the hippocampus of aged mice may alleviate cognitive impairment induced by anesthesia and surgery. This observation implies an association between the reduction of glutamatergic synapses and cognitive dysfunction in aged mice.

In the present study, we employed snRNA‐seq technology to examine the alterations of excitatory neurons in the hippocampus associated with long‐term cognitive impairment induced by sevoflurane in aged mice. We conducted a comprehensive analysis of DEGs in excitatory neurons using GO and KEGG methodologies, investigating the impact of sevoflurane anesthesia on excitatory neurons in aged mice. Subsequently, validation was performed through immunofluorescence staining of glutamatergic synapses in the hippocampus of aged mice. The findings of our study would offer valuable insights into the role of excitatory neurons in neurodegenerative conditions induced by sevoflurane anesthesia.

## 2 Materials and Methods

### 2.1 Anesthesia of Aged Mice

The mouse experiments were performed in accordance with the procedures approved by the Animal Care and Use Institution Committee of Shanghai Ninth People's Hospital (SH9H‐2021‐A831‐1). We used 18‐month‐old C57/6 J male mice obtained from Beijing Vital River Experimental Animal Technology Co. Ltd. The mice were housed under specific pathogen‐free conditions, with ambient parameters set at a temperature of 25°C–27°C, humidity ranging from 30% to 70%, and a 12‐h light/dark cycle. The animals were provided with free access to water and feed.

Aged mice were randomly divided into the sevoflurane (SEV) or control (Ctrl) groups. In the SEV group, aged mice were exposed to 2% sevoflurane and 60% O_2_ (at a flow rate of 1 L/min) for 6 h, while the mice in the Ctrl group were exposed to 60% O_2_ alone (at a flow rate of 1 L/min) for the same duration. As described in our previous study, we monitored the blood levels of electrolytes, including sodium, potassium, and chloride to ensure they remained within the normal range [10]. The monitored heart rate and saturation of pulse oxygen (SpO_2_) were consistent with normal physiological parameters. Importantly, no hypoxia or carbon dioxide accumulation was observed throughout the monitoring period. During general anesthesia, spontaneous respiration was maintained in the mice, with strict monitoring, to ensure that their rectal temperature remained within the range of 37°C ± 0.5°C. In our study, a total of 18 mice were used, with six designated for snRNA‐seq analysis and the remaining 12 utilized for immunofluorescence staining. After anesthesia or oxygen inhalation, all aged mice were euthanized. For snRNA‐seq analysis, the hippocampus of mice in Ctrl and SEV groups (*n* = 3) were quickly removed and preserved in liquid nitrogen. For immunofluorescence, the hippocampus of mice in Ctrl and SEV groups (*n* = 6) were quickly removed and post‐fixed in 4% paraformaldehyde at 4°C.

### 2.2 snRNA‐Seq Analysis

#### 2.2.1 Nucleus Isolation

The samples were surgically removed and the frozen tissue was used to intact nucleus isolation. The nucleus was isolated and purified as previously described with some modifications [11]. Briefly, the frozen tissue was homogenized in NLB buffer, which contains 250 mM Sucrose, 10 mM Tris–HCl, 3 mM MgAc2, 0.1% Triton X‐100 (SigmaAldrich, USA), 0.1 mM EDTA, and 0.2 U/μL RNase Inhibitor (Takara, Japan). Various concentrations of sucrose were used to purify the nucleus.

#### 2.2.2 Single‐Nuclei RNA Sequencing

The snRNA‐Seq libraries were generated using the 10× Genomics Chromium Controller Instrument and Chromium Single Cell 3' V3.1 Reagent Kits (10× Genomics, Pleasanton, CA). Briefly, nuclei were concentrated to approximately 1000 nuclei/μL and loaded into each channel to generate single‐cell gel bead‐in‐emulsions (GEMs). After the RT step, GEMs were broken and barcoded‐cDNA was purified and amplified. The amplified barcoded cDNA was fragmented, A‐tailed, ligated with adaptors, and index‐PCR amplified. The final libraries were quantified using the Qubit High Sensitivity DNA assay (Thermo Fisher Scientific), and the size distribution of the libraries were determined using a High Sensitivity DNA chip on a Bioanalyzer 2200 (Agilent). All libraries were sequenced by the Illumina sequencer (Illumina, San Diego, CA) on a 150 bp paired‐end run.

#### 2.2.3 Statistical Analysis of snRNA‐Seq

snRNA‐seq data analysis was performed by NovelBio Co. Ltd. with NovelBrain Cloud Analysis Platform (www.novelbrain.com). We applied fastp with default parameter filtering the adaptor sequence and removed the low quality reads to achieve the clean data. Then the feature‐barcode matrices were obtained by aligning reads to the rhesus mouse genome (mm10 Ensemble: version 100) using CellRanger v6.1.1 with intron including mode for the data characteristics of snRNA‐Seq and utilized the cellranger aggr function with mapped reads. We modified and achieved the aggregate expression matrix for further analysis. Seurat package (v4.0.3) was used for cell filtering (mito% < 0.1; 200 < gene num < 10,000), dimension reduction to construct PCA, UMAP and tSNE (variable feature = 2000; Max PC =50; PC use = top10 significant PC); and graph clustering (PC use = 1:10, resolution = 0.8). To calculate the marker genes of each cluster, we applied the Findmarkers function with wilcox ranksum test following the criteria: (1) lnFC > 0.25; (2) *p* < 0.05; (3) min.pct > 0.1. Based on the specific marker (pct1‐pct2 > 0.2; FC > 0.5; FDR < 0.01), we identified the cell type of each cluster according to the known marker in snRNA‐Seq such as AQP4 for astrocyte, P2RY12 for microglia, PDGFRA for OPC, GAD1 for interneuron, SLC17A6/7 for excitatory neurons MBP/MOG/PLP1 for oligodendrocytes. Moreover, we combine the cluster with same cell type for visualization and applied the sub‐clustering analysis for each cell type.

#### 2.2.4 Pseudo‐Time Analysis

We applied the trajectory analysis utilizing Monocle2 (http://cole‐trapnell‐lab.github.io/monocle‐release) using DDR‐Tree and the default parameter. Before monocle analysis, we select marker genes of the Seurat clustering result and raw expression counts of the cell passed filtering. Based on the pseudo‐time analysis, branch expression analysis modeling (BEAM Analysis) was applied for branch fate determined gene analysis.

#### 2.2.5 Gene Ontology Analysis

Gene Ontology analysis was performed to facilitate elucidating the biological implications of marker genes and DEGs. We downloaded the GO annotations from NCBI (http://www.ncbi.nlm.nih.gov/), UniProt (http://www.uniprot.org/), and the GO (http://www.geneontology.org/). Fisher's exact test was applied to identify the significant GO categories, and FDR was used to correct the *p*‐values.

#### 2.2.6 Pathway Analysis

Pathway analysis was used to find out the significant pathway of the marker genes and DEGs according to the KEGG database. We turn to the Fisher's exact test to select the significant pathway, and the threshold of significance was defined by *p*‐value and FDR.

### 2.3 Immunofluorescence

Mice were deeply anesthetized using an overdose of sevoflurane and transcardially perfused with PBS, followed by 4% paraformaldehyde in PBS. Brains were removed and post‐fixed in 4% paraformaldehyde at 4°C for 24 h. Immunofluorescence was performed on 30 μm thick cryosectioned brain slices. Free‐floating sections were washed with PBS (three times, 8 min each), rinsed, and then transferred to blocking buffer (10% normal goat serum, 0.3% Triton X‐100 in PBS) for 1 h at 37°C. After blocking, the sections were incubated with mouse anti‐PSD95 (1:100, Cell Signaling Technology, 36233) and rabbit anti‐vGLUT1 (1:100, Abcam, ab227805). Following PBS washes (3 times, 10 min each), Alexa 488 (abcam; ab150077; 1:500) and Alexa 594 (abcam; ab150116; 1:500) were then used to conjugate the secondary antibody at 37°C for 2 h. The slices were stained with DAPI for 10 min at room temperature (25°C ± 2°C), washed with PBS (3 times, 10 min each), and mounted in 70% glycerol. Finally, the images were taken with confocal microscopy (A1, Nikon, Japan). Images of sections containing the target brain regions were cropped and analyzed using ImageJ.

### 2.4 Statistics

We validated that each data set follows a normal distribution using the Shapiro–Wilk test. All values were presented as mean ± standard error of the mean (SEM). The data were analyzed by one‐way ANOVA analysis of variance followed by the Tukey's test. GraphPad Prism 9.0 (GraphPad, USA) software was used for statistical analysis. Statistical significance was accepted as *p < 0.05.

## 3 Results

### 3.1 Hippocampus Single‐Nucleus Transcriptome of Aged Mice

**FIGURE 1:**
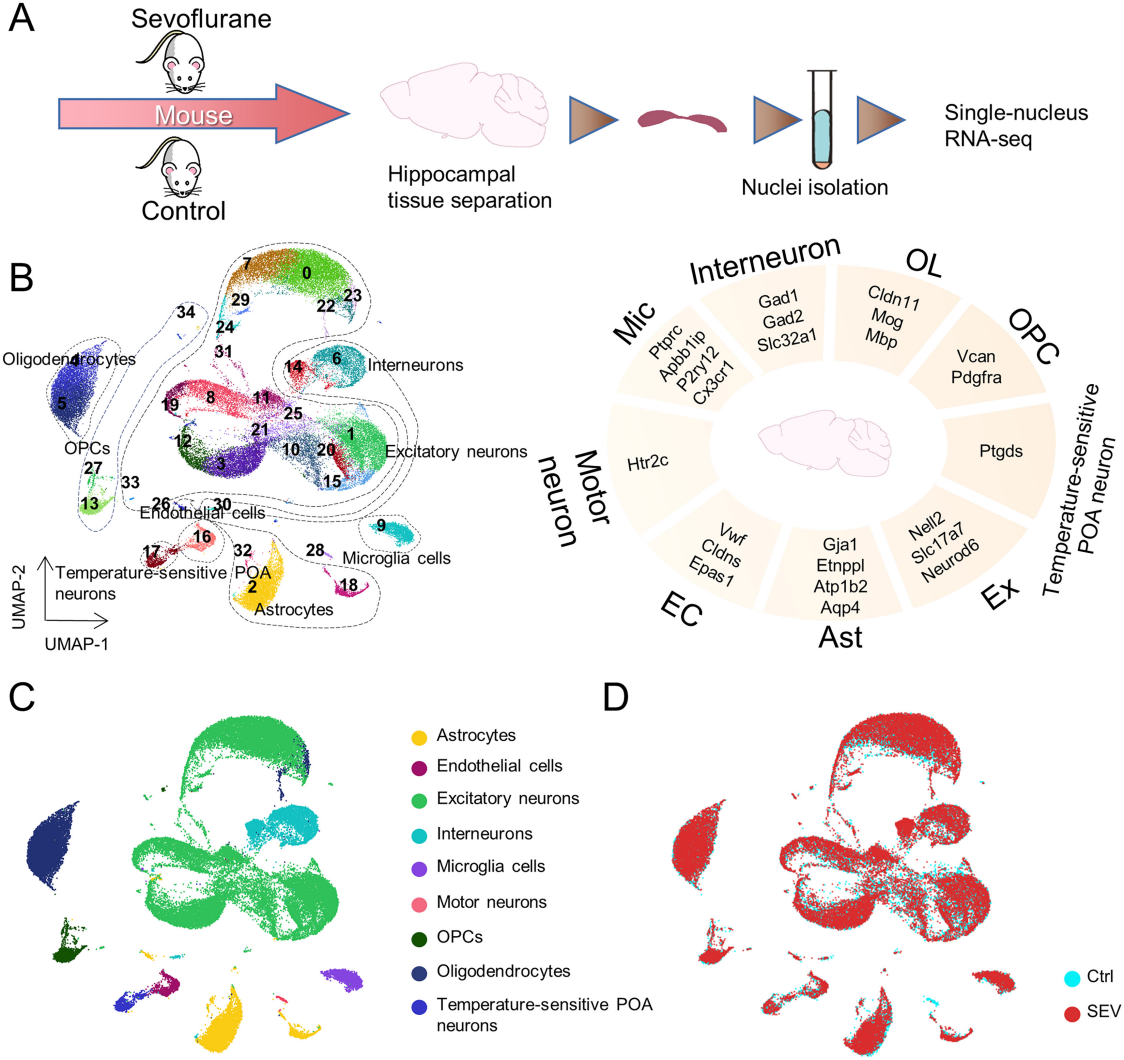
Hippocampus single‐nucleus transcriptome of aged mice. (A) Schematic graph showing the experimental design and general workflow of snRNA‐seq, including three key steps. (1) Three mice were in the control group (Ctrl). Three mice were in the sevoflurane group (SEV). (2) Hippocampus was dissected from mouse and harvested according to the Allen Brain Atlas. Each sample was used to obtain single‐nucleus suspensions. (3) Nuclei isolation was performed and snRNA‐seq was finally measured and analyzed. (B) Different snRNA‐seq markers representing different cell types, including excitatory neurons (Ex), astrocytes (Ast), endothelial cells (EC), motor neuron, microglia (Mic), interneurons, oligodendrocytes (OL), oligodendrocyte precursor cells (OPC), Temperature‐sensitive POA neurons of mouse. (C, D) UMAP representation of diverse cell types of mice, were identified by the specific snRNA‐seq marker and colored in cluster (C) or group (D).

To understand the molecular features of aged mice's hippocampus, we analyzed cells from the mouse hippocampus using snRNA‐seq visualized in UMAP. Schematic graph showing the experimental design and general workflow of snRNA‐seq, including three key steps. We collected the nuclei cells from the hippocampus of mice and performed snRNA‐seq using samples combined from six individual mice (Figure 1A). Distinct snRNA‐seq markers representing various cell types, including excitatory neurons (Ex, whose markers are Nell2, Slc17a7, and Neurod6), astrocytes (Ast, whose markers are Gja1, Etnppl, Atp1b, and Aqp4), endothelial cells (EC, whose markers are Vwf, Cldns, and Epas1), motor neuron (whose marker is Htr2c), microglia (Mic, whose markers are Ptprc, Apbb1ip, P2ry12, and Cx3cr1), interneurons (whose markers are Gad1, Gad2, and Slc32a1), oligodendrocytes (OL, whose markers are Cldn11, Mog, and Mbp), oligodendrocyte precursor cells (OPC, whose markers are Vcan and Pdgfra), temperature‐sensitive POA neurons (whose marker is Ptgds) of mouse (Figure 1B). Moreover, UMAP visualization was employed to portray a comprehensive representation of varied cell types in mice. These cell types were identified by the specific snRNA‐seq markers and were differentiated by their distinct coloring in clusters (Figure 1C) or groups (Figure 1D). From Figure 1C,D, it is evident that excitatory neurons represent the most abundant neuronal cell type in the hippocampus of aged mice. In snRNA‐seq, we employed samples from three aged mice to identify trends. Further validation will be conducted by immunofluorescence staining. Finally, we employed UMAP to broadly construct the transcriptome and understand the molecular features of the aged mouse hippocampus using snRNA‐seq.

### 3.2 Excitatory Neuron Cluster Taxonomy of Mice's Hippocampus

**FIGURE 2:**
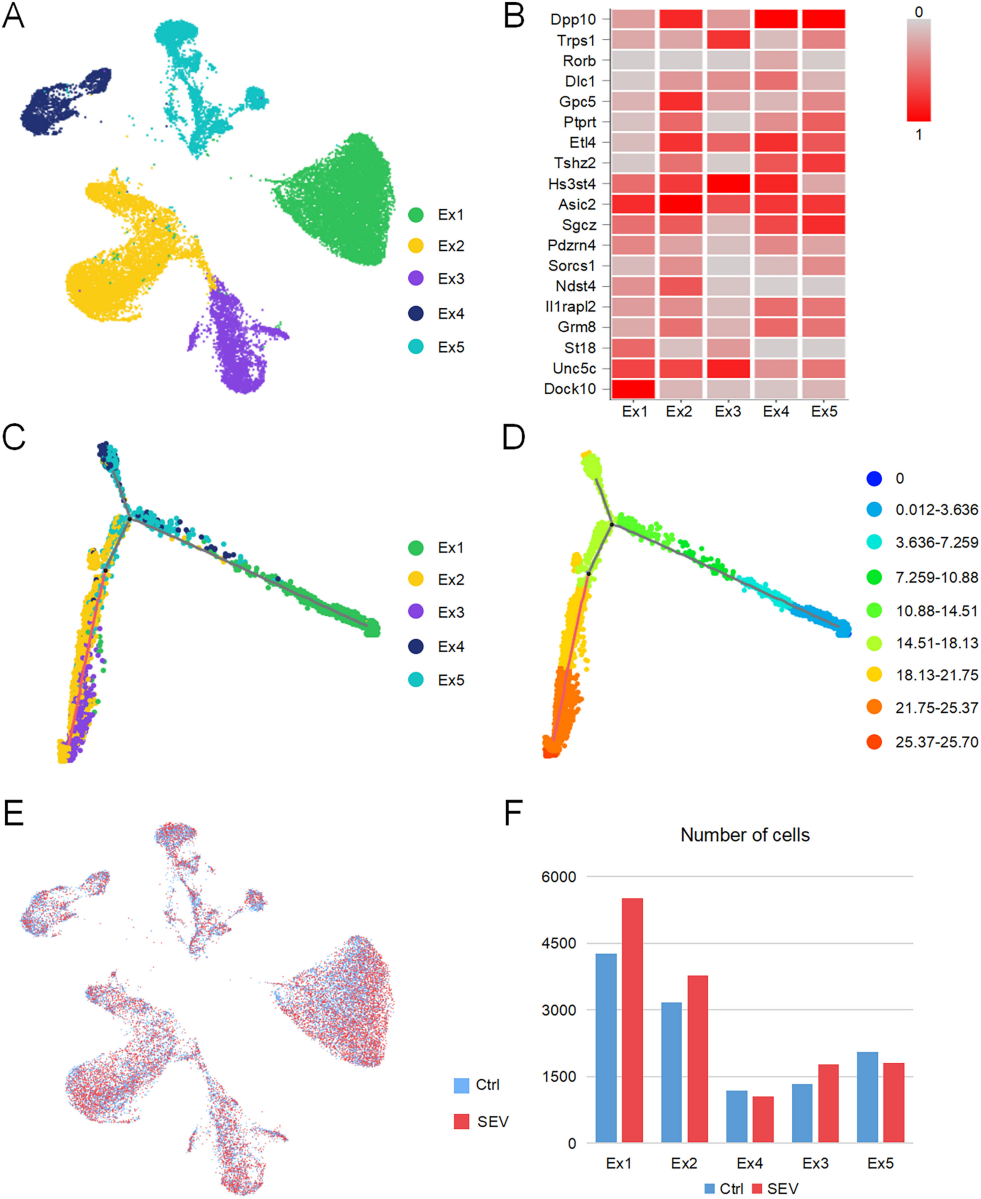
Excitatory neuron cluster taxonomy of mice's hippocampus. (A) UMAP representation of excitatory neurons colored by cluster. (B) Heatmap of 20 snRNA‐seq marker genes in excitatory neurons of mouse. (C) The scatter plot shows the distribution of five distinct excitatory neuron clusters. Each point represents a single cell, colored by its assigned subtype. (D) Cells are colored based on their calculated stemness score, with a gradient ranging from high stemness (red) to low stemness (blue). (E) UMAP representation of excitatory neurons colored by group (red, SEV; blue, Ctrl). (F) Bar plot showing the differences in mouse excitatory neuron cluster between group SEV and Ctrl. The number of excitatory neurons following Ctrl are close to the number of excitatory neurons following SEV in each cluster of mice.

To further investigate the characteristics of hippocampal neurons in aged mice, we subclassified all the excitatory neurons of the hippocampus into five clusters by UMAP. In Figure 2A, excitatory neurons were categorized into five clusters. Cells were colored based on their respective clusters (Figure 2A). We presented a heatmap displaying 20 selected snRNA‐seq marker genes within the excitatory neurons of mice (Figure 2B). Pseudo‐time analysis was carried out to examine if these five clusters have any internal relationship. Our results showed a distinct hierarchical order of stemness across the clusters, with cluster 3 showing the highest stemness, followed by clusters 2, 5, 4, and 1 (Figure 2C,D). The significance of this stemness gradient likely reflects the functional diversity and adaptability of these neurons in the aged brain. Cluster 3, with the highest stemness, may represent a population with higher plasticity or regenerative potential, contributing to the brain's capacity for repair or adaptation in response to aging‐related stressors. In contrast, cluster with lower stemness, such as cluster 1, might be more terminally differentiated, playing stable and specialized roles in hippocampal function. The progression from cluster 3 to 2, followed by 5, 4, and finally 1 (Figure 2C,D), suggested that these subtypes were not isolated entities, but rather represent different stages along a common differentiation pathway.

While in Figure 2E, cells were colored by groups, with red representing the SEV group and blue representing the Ctrl group (Figure 2E). This enabled a meticulous examination and identification of specific neurobiological patterns or distinctive features within the aged hippocampal excitatory neurons. A bar plot illustrated distinctions in the mouse excitatory neuron cluster between the SEV group and Ctrl group (Figure 2F). The bar plot showed that after sevoflurane anesthesia, the excitatory neuron counts in the Ctrl group closely approximate those in the SEV group within each mouse cluster. This observation highlights the comparable population sizes of excitatory neurons between the two experimental conditions, suggesting that sevoflurane might not induce cognitive dysfunction by altering the neuron count of various subtypes within the excitatory neurons. It emphasized the need for further quantitative and qualitative analyses to illustrate potential alterations in the molecular and functional profiles of these neurons. Therefore, conducting a more thorough exploration of the DEGs within each excitatory neuron cluster is deemed essential.

### 3.3 Sevoflurane Anesthesia Caused DEGs in Mouse

**FIGURE 3:**
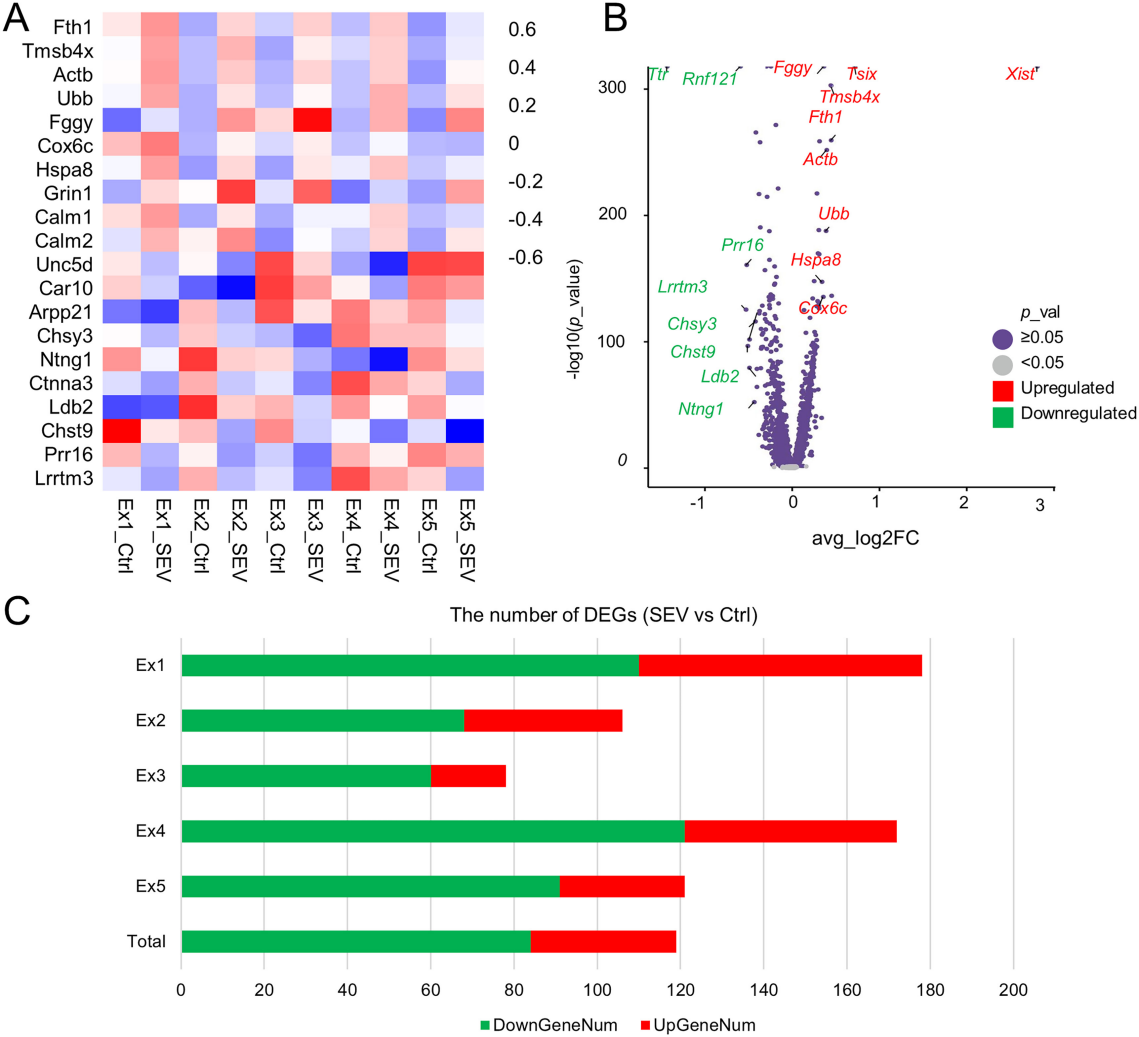
Sevoflurane anesthesia caused DEGs in mouse. (A) Heatmaps representation of 20 DEGs in excitatory neuron clusters of mice's hippocampus after sevoflurane exposure following Ctrl versus SEV. Demonstration of the heatmap indicates that sevoflurane anesthesia caused diverse changes in mouse. (B) Volcano plots representation of DEGs in excitatory neuron of mouse after sevoflurane exposure. In the volcano plot, *p* < 0.05 was set as the cut‐off criterion of significant difference. Red tags representation of the up‐regulated significant DEGs and green tags representation of the downregulated significant DEGs. (C) Bar graph showing the number of DEGs in excitatory neurons clusters of mice after sevoflurane exposures between Ctrl and SEV group. Red bars illustrating the number of up‐regulated genes and green bars representing the number of downregulated genes.

In distinct excitatory neuron clusters, we conducted DEGs analysis and represented the results using a heatmap and volcano plot. Visualization of the heatmap reveals alterations in gene expression induced by sevoflurane anesthesia in mice (Figure 3A). The volcano plot illustrates the top snRNA‐seq marker genes in aged mouse excitatory neurons (Figure 3B). From the figures, it is evident that the genes Fth1, Tmsb4x, Actb, Ubb, Fggy, Cox6c, Hspa8, Grin1, Calm1, and Calm2 exhibit an upregulation trend, whereas the genes Unc5d, Car10, Arpp21, Chsy3, Ntng1, Ctnna3, Ldb2, Chst9, Prr16, and Lrrtm3 demonstrate a downregulation trend.

Furthermore, we quantified the number of DEGs in mouse excitatory neuron clusters after sevoflurane exposure compared to the Ctrl group, visualized in the bar graph (Figure 3C). From the bar graph, it was evident that excitatory neuron cluster 1 exhibits the highest number of upregulated and downregulated genes after sevoflurane exposure, followed by excitatory neuron cluster 4. In contrast, excitatory neuron cluster 3 showed the least number of both upregulated and downregulated genes after sevoflurane exposure. In summary, sevoflurane induced more downregulation of genes in excitatory neurons of aged mice compared to upregulated genes. However, the implications represented by the upregulation and downregulation of these genes still need further investigation.

### 3.4 Significant Enriched GO and KEGG Pathway Terms of DEGs in Mice's Hippocampus After Sevoflurane Anesthesia

**FIGURE 4:**
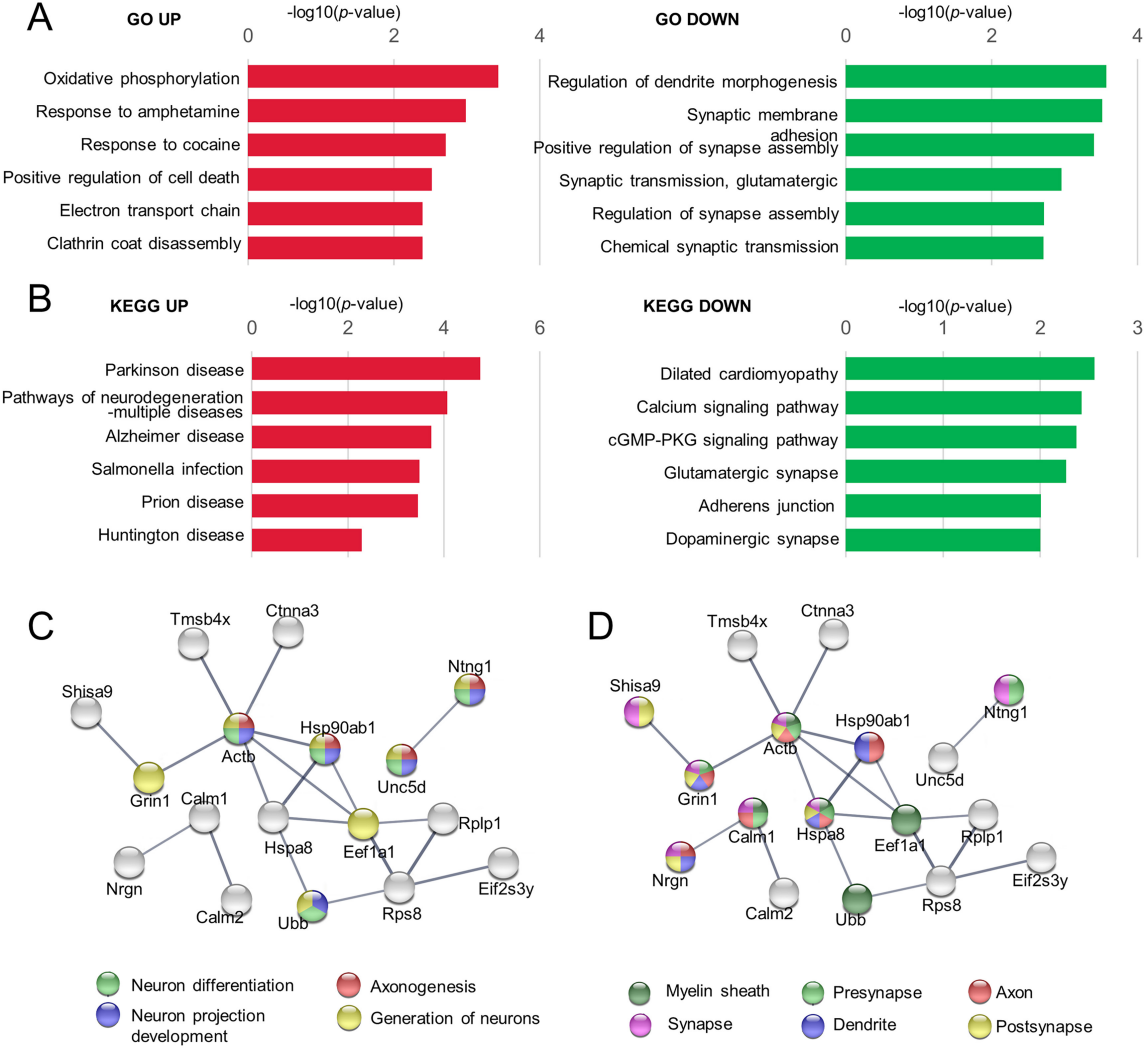
GO and KEGG pathway terms of DEGs in mice's hippocampus after sevoflurane anesthesia. (A) GO enrichment of DEGs in excitatory neuron of mouse, illustrating the biological functions of mouse. Bars colored by red demonstrating the GO enrichment of up‐regulated DEGs and green demonstrating the GO enrichment of downregulated DEGs. (B) Bar plots showing the KEGG enrichment in excitatory neuron of mouse. Red bars illustrating the enriched KEGG pathways of up‐regulated DEGs and green bars representing the enriched KEGG pathways of downregulated DEGs. Significant enriched biological process (gene ontology) (C) and cellular component (gene ontology) (D) terms of the DEGs (*p* < 0.05, FC > 1.2 or FC < 0.8) in excitatory neurons of mice's hippocampus after sevoflurane anesthesia.

To further explore the biological functions of DEGs in excitatory neurons of mouse, we conducted GO and KEGG analyses. Bars colored in red indicate the upregulated genes, while those colored in green represent the downregulated genes. We observed a decline in functions associated with synapses in excitatory neurons after sevoflurane exposure, including downregulation of synapse assembly, glutamatergic synaptic transmission, and chemical synaptic transmission (Figure 4A). Bar plots of KEGG enrichment in excitatory neurons of mice showed the upregulation of Alzheimer disease, other neurodegenerative multiple diseases, and downregulation of glutamatergic synapse (Figure 4B). Significantly enriched biological process and cellular component terms of the DEGs in excitatory neurons also showed association with synaptic functions (Figure 4C,D).

Synapses are basic information processing units in the brain [6]. The correct number and type of synaptic connections are essential for normal brain functions. Decreased hippocampal glutamatergic synapse density is associated with cognitive dysfunction [8]. A previous study has indicated that sevoflurane anesthesia may result in PND [10]. The results of functional analysis of the DEGs in the previous figure represented a decrease in genes Ntng1 and Lrrtm3 due to sevoflurane anesthesia. Notably, both Ntng1 and Lrrtm3 are associated with the synaptic function of excitatory neurons [19]; [18]. Previous studies showed that Ntng1 is implicated in neuronal circuit formation at synaptic levels, while Lrrtm3 is involved in presynapse assembly [18, 19]. The density of hippocampal glutamatergic synapses is associated with cognition in rodents [6]. Downregulation of Ntng1 and Lrrtm3 might affect hippocampal glutamatergic synapses and contribute to age‐related cognitive impairment, which is consistent with our functional analysis results.

In our study, we observed a decline in functions associated with synapses in excitatory neurons after sevoflurane exposure. These results indicated that the reduction of glutamatergic synapse may represent one of the potential mechanisms.

### 3.5 Sevoflurane Downregulates the Levels of PSD95 and vGLUT1 in the Hippocampus of Aged Mice

**FIGURE 5:**
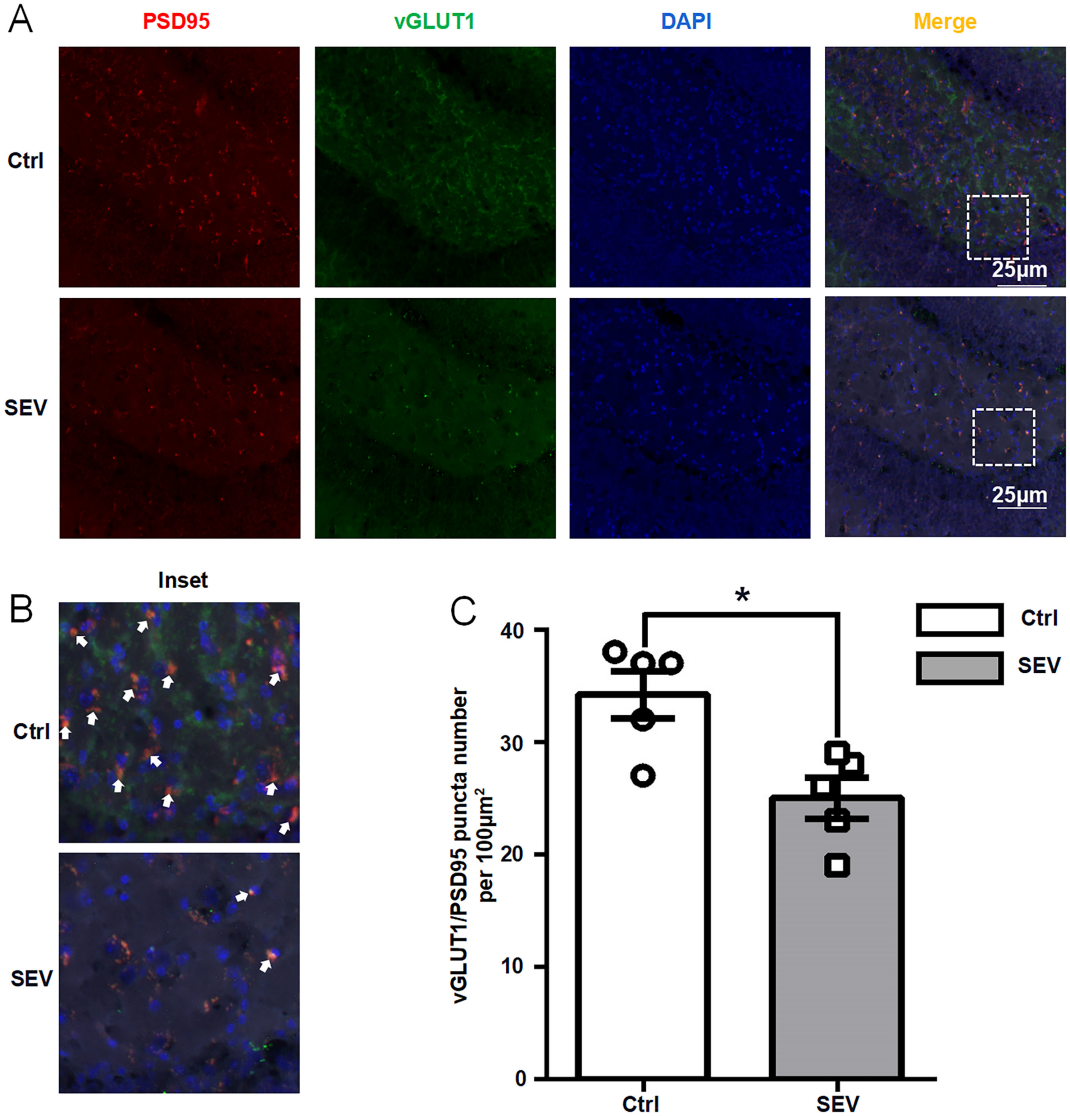
Sevoflurane downregulates the levels of PSD95 and vGLUT1 in the hippocampus of aged mice. (A, B) Glutamatergic synapses were stained by immunofluorescence (PSD95‐red, vGLUT1‐green, DAPI‐blue, merged image‐yellow) in the hippocampus of aged mice. (C) Bar plot of vGLUT1/PSD95 puncta number per 100 μm. Data are expressed as the mean ± SEM (one‐way ANOVA followed by Bonferroni's post hoc test, *n* = 6 per group). *p < 0.05 versus he Ctrl group.

The synaptic density within the hippocampus is associated with cognitive functions in rodents [8]. The co‐localization assessment of postsynaptic density protein 95 (PSD95) and vesicular glutamate transporter 1 (vGLUT1) is widely used to specifically label the glutamatergic synapses in neurobiological investigations [6]. Therefore, we investigated the correlation between sevoflurane anesthesia and hippocampal glutamatergic synapse density and subsequently performed a statistical analysis. Immunofluorescence staining of glutamatergic synapses was conducted in the hippocampus of aged mice (Figure 5A,B). The statistical results revealed a notable reduction in the density of glutamatergic synapses within the hippocampus after sevoflurane anesthesia in aged mice (Figure 5C). These results indicated that sevoflurane might decrease the density of glutamatergic synapses in the hippocampus.

## 4 Discussion

PND, including POD and POCD, are common following anesthesia and surgery in older patients and might affect memory, consciousness, information processing, and social ability [12]. These disorders may deteriorate functional outcomes and long‐term quality of life after surgery, thereby resulting in a substantial social and financial burden to both the family and society [4]. Previous studies have indicated that surgery/anesthesia can induce cognitive decline in both human patients and rodents [6]. In our study, aged mice were exposed to sevoflurane, and snRNA‐seq was performed, which enables the acquisition of high‐resolution gene expression data. Using snRNA‐seq technology to map the brain transcriptome of aging mice facilitates the further exploration of anesthesia mechanisms. Currently, UMAP is widely utilized for classification in numerous studies, exhibiting broader and superior applicability compared to t‐SNE [13]. Therefore, we chose UMAP to visualize the data from aged mice in our study. After GO and KEGG enrichment analyses, we observed that sevoflurane anesthesia might lead to neurodegenerative multiple diseases. However, the mechanism of cognitive dysfunction is rather complicated.

The balance between excitatory and inhibitory neurons within the central nervous system is essential for maintaining proper brain function [14]. Previous studies have shown that sevoflurane might lead to an imbalance between excitatory and inhibitory neurons [15]. Excitatory neurons, a prominent neuronal subtype within the brain and essential components of the central nervous system, play a crucial role in modulating neural circuitry and governing cognitive functions [5]. Our study suggested that sevoflurane anesthesia did not alter the number of excitatory neurons. However, it may induce dramatic changes in their gene expression profiles. After GO and KEGG pathway analyses of DEGs in excitatory neurons, we observed a significant modulation in synaptic functions within excitatory neurons after sevoflurane exposure, including downregulation of crucial processes such as synapse assembly, glutamatergic synaptic transmission, and chemical synaptic transmission. Furthermore, KEGG enrichment analysis revealed an upregulation of pathways associated with Alzheimer's disease and other neurodegenerative conditions, alongside a downregulation of genes linked to glutamatergic synapse functions in excitatory neurons of aged mice. These findings suggest a potential involvement of excitatory neurons in the observed cognitive dysfunction in aged mice.

The principal synaptic type of excitatory neurons comprises glutamatergic synapses [6]. Glutamatergic synapses serve as fundamental information processing units in the brain, which are essential for normal central nervous system functions by ensuring the accurate number and type of synaptic connections [16]. In particular, glutamatergic synapses play a crucial role in critical cognitive processes such as memory formation, learning, and emotional regulation. A previous study by Xiong et al. [8] established a link between a reduced density of hippocampal glutamatergic synapses and cognitive impairment in rats with neuropathic pain. Building upon this knowledge, our investigation in aged mice revealed that sevoflurane downregulated the levels of presynaptic vGLUT1 and postsynaptic PSD95, markers of excitatory synapses. This suggests that sevoflurane anesthesia might impair cognition by inhibiting glutamatergic synaptogenesis within the hippocampus. Our investigation into the synapses of hippocampal glutamatergic neurons has provided a potential therapeutic target for preventing the sevoflurane‐induced PND in the elderly.

This study has several limitations. Firstly, our snRNA‐seq results only provide a snapshot of excitatory neuron changes at a specific time after sevoflurane exposure. However, it still offers a high‐resolution view, providing valuable insights into the aged brain. To address this, future studies could collect samples at various time points. Secondly, the study focuses on long exposure to anesthesia in aged mice, neglecting short exposure effects. However, long exposure to anesthesia is clinically significant due to its association with increased postoperative complications. Thirdly, our study has a limitation in the absence of behavioral test validation. Despite this, GO and KEGG analyses partially support the observed cognitive decline. Future studies would include behavioral tests, like the Morris water maze, to more precisely evaluate sevoflurane anesthesia‐induced cognitive impairment. Lastly, the mouse model may not fully replicate the complex pathology of human PND. Future investigations using NHPs could better replicate the intricate pathology associated with human PND, building upon our previous study with young rhesus macaques [17].

## 5 Conclusions

In conclusion, this study demonstrates that DEGs in excitatory neurons are associated with reduction of glutamatergic synapses and cognitive dysfunction. Through immunofluorescence staining validation, we also confirmed that sevoflurane anesthesia decreased the density of glutamatergic synapses in the hippocampus of aged mice. These results demonstrate a key role of hippocampal glutamatergic synaptic alterations in sevoflurane‐induced cognitive dysfunction in aged mice.

## Author Contributions

Hong Jiang, Jia Yan, Lei Zhang, Yixuan Niu, and Guoying Liao initiated and designed the study. Yixuan Niu, Guoying Liao, Zhengjie Miao, Jinnan Xu, Yanyong Cheng, Lei Zhang, Fan Wang, Chuanyu Qi, Tiannan Chen, and Yi Gao prepared the material and methods. Yixuan Niu, Guoying Liao, Zhengjie Miao, and Jinnan Xu conducted immunofluorescence experiments. Yixuan Niu and Zhengjie Miao performed the sample collection and data analysis. All authors approved the manuscript prior to submission.

## Conflicts of Interest

The authors declare no conflicts of interest.

## Data Availability

The data that support the findings of this study are openly available in Gene Expression Omnibus (GEO) at https://www.ncbi.nlm.nih.gov/geo/query/acc.cgi, reference number GSE234493.
